# Will Happiness Improve the Psychological Integration of Migrant Workers?

**DOI:** 10.3390/ijerph15050900

**Published:** 2018-05-03

**Authors:** Tian-Cheng Li, Chien-Chi Chu, Fan-Cun Meng, Qin Li, Di Mo, Bin Li, Sang-Bing Tsai

**Affiliations:** 1 College of Economics and Management, South China Agricultural University, Guangzhou 510642, China; lycmfc@163.com (T.-C.L.); liqin@scau.edu.cn (Q.L.); 2 Department of Finance, Business School, Shantou University, Shantou 515063, China; 3 Research Institute for Guangdong-Taiwan Business Cooperation, Shantou University, Shantou 515063, China; 4 School of Economics, Finance and Marketing, College of Business, RMIT University, Melbourne, VIC 3000, Australia; di.mo@rmit.edu.au; 5 Department of Accounting, Finance and Economics, Griffith University, Brisbane, QLD 4111, Australia; b.li@griffith.edu.au; 6 Zhongshan Institute, University of Electronic Science and Technology of China, Guangzhou 528400, China; 7 Research Center for Environment and Sustainable Development of China Civil Aviation, Civil Aviation University of China, Tianjin 300300, China

**Keywords:** migrant workers, happiness, psychological integration, psychological health

## Abstract

Happiness is a major factor that influences people’s perceptions and behavior. Two-stage least squares regression was applied to investigate the effect of happiness on the psychological integration of migrant workers in China. The data for a total of 1625 individuals were obtained from the 2014 China Labor-force Dynamics Survey (CLDS). This study describes happiness from three main aspects: happiness, life satisfaction, and economic satisfaction. The psychological integration includes two dimensions of settlement willingness, and trust level; these have gone through dimension-reduced processing by using the weighted average method. The empirical evidence shows, first, that happiness has a significantly positive effect on the psychological integration of migrant workers and second, that the sense of life satisfaction in particular plays a more significant role. The acceleration of the social and political integration in migrant workers will enhance their psychological integration. Additionally, social, cultural and economic integration is found to influence migrant workers’ psychological integration by promoting happiness. Happiness between different generations of migrant workers was found to have a noticeably positive impact on their psychological integration; however, the happiness of the younger migrant workers was more perceivable than that of the other generations. Preferential policies should therefore be provided to improve the happiness of migrant workers.

## 1. Introduction

One of the most prominent changes in Chinese society since the Chinese government implemented the reform and opening-up policy in 1978 has been the unprecedented population movement. This is also one of the main driving forces of social and population change [1]. According to a National Bureau of Statistics survey, the total number of migrant workers in 2015 was 277 million, out of which 168 million were migrant workers and 109 million were local migrant workers (National Migrant Worker Monitoring Survey Report 2015, National Bureau of Statistics, http://www.gov.cn/xinwen/2015-04/29/content_2854930.htm. ‘Local migrant workers’ indicates workers who stay in their county; ‘migrant workers’ are those who migrate across counties). Migrant workers are considered to be a group that has been marginalized and made vulnerable by society and, as a result, their degrees of urban adaptation and life satisfaction are low. After entering cities, they gradually experience the change from physiology and society to culture. Most of the migrant workers, however, are dissociated from the edge of urban society and rural society [2]. They are facing the dilemma of integrating into urban society. China is currently undergoing a new stage of development, the “One Belt and One Road”, which is a critical period of integration between urban and rural areas. The result of assimilating the floating population into urban society has attracted the growing attention of the academic community [3,4,5]. With the continuous development of urbanization to ensure migrant workers can become urban citizens in China, the sense of belonging, in the context of the substantial environmental differences, has become an important factor in the measurement of migrant “homogenization” with urban residents. True urban integration involves migrant workers developing a high level of psychological identification with their new destinations [6]. Only when migrant workers perceive with a high level of psychological identification can they truly be integrated into city life. Furthermore, the sense of happiness and satisfaction of a migrating population at their destination can be considered as a predictor of their intention to undertake long-term residence at that destination. Surveys showed that the level of happiness perceived by rural and urban residents is much higher than that perceived by migrant workers [7]. Happiness is a major factor that influences individuals’ perceptions and behavior. However, no adequate attention from either local governments or scholars has investigated the impact of migrant workers’ perceived well-being on their urban integration. Several questions remain unsolved: for example, the current status of migrant workers’ psychological integration when faced with a disorienting and fast-paced urban life; the impact of migrant workers’ perceived happiness on their psychological integration; the influences of other dimensions of urban integration on migrants’ psychological integration and how these make an impact; and intergenerational differences in the impact of migrant workers’ perceived happiness on psychological integration. Findings in relation to these issues are of great importance for appropriate urban integration of migrant workers and for the promotion of new urbanization.

In this paper, we use the data of the 2014 China Labor-Force Dynamics Survey (CLDS), provided by the Center for Social Survey at Sun Yat-sen University, to investigate the impact of happiness on the urban integration of migrant workers at a psychological level, from the perspective of social economy. We find that the happiness, life satisfaction, and economic satisfaction of migrant workers have a significant impact on the level of the psychological integration of migrant workers. Life satisfaction has a greater impact than economic satisfaction. In addition, social and political integration has a significant impact on the level of the psychological integration of migrant workers. Furthermore, the economic, social and cultural integrations influence psychological integration by affecting the happiness of migrant workers.

Our study makes two main contributions. First, using Chinese data as its sample, it contributes to Happiness Economics research by providing further evidence regarding the effect of happiness on the psychological perception and behaviors of residents. Second, our study provides an innovative perspective regarding the impact of happiness on psychological integration of migrant workers during the process of their urban integration, while past studies mostly focus on the source and determinants of the happiness of residents.

The remainder of this paper is organized as follows. Section 2 provides a brief literature review, and Section 3 develops our research hypotheses. Section 4 contains data source and research design, while Section 5 provides variable definition and basic descriptive statistics of variables. Section 6 reports our empirical results, and Section 7 provides robustness checks. Finally, Section 8 concludes our paper and provides some policy recommendations.

## 2. Literature Review

The concept of urban integration is derived from one of the social integrations. In modern society, urban integration for migrant workers is an issue faced in every country. As early as 1890, Park from the Chicago School conducted research on the social integration of immigrants living in the United States [8]. Since then, scholars have conducted many in-depth studies with respect to aspects of social integration, such as definition [9,10], dimensions [11], strategies [12,13], and influencing factors [9,14,15]; consequences of social integration [16], such as the cultural context, may influence maternal experience and behaviors relevant to infant weight and growth [17].

The existing literature has identified many dimensions of social integration, such as Gordon’s Seven Dimensions Theory, Alba and Nee’s extension of Gordon’s theory with social and economic integration dimensions, Massey’s Living Integration Dimension, and Maxwell’s Political Integration [9,10,18,19]. In China, large numbers of migrant workers have continuously been flowing into cities since the 1980s. Scholars in China have introduced the concept of “urban integration” in their research on migrant workers. Notable among their theories are Zhu’s Three Dimensions Theory, Yang’s Four Dimensions Theory, and Zhang and Lei’s Four Dimensions Theory [20,21,22].

The common integration dimensions contain objective (e.g., economy, society, culture) and subjective (such as psychological identity) levels [15,23,24], but the objective level is involved in some of this research only [6,24]. Thus far, scholars have not yet reached a consensus regarding which are the best dimensions of the social integration of migrant workers to focus on. Our extensive review of the literature leads us to argue that the dimensions of urban integration include the integration of migrant workers in aspects of their economic life, their social interaction, their political activities, their cultural exchanges, and their psychological cognition. These dimensions, however, are not simply linearly related to each other; rather, they are mutually blended and infiltrated. They probably start at the same time but end with varying degrees of integration.

Factors that influence the urban integration of migrant workers have always been a popular area of academic research. Chinese scholars have worked on aspects such as human capital, social capital, and social protection in order to determine these influencing factors. Changing terminology from “migrant worker” to “urban citizen” does not indicate that migrant workers have psychologically integrated into urban society. Psychological integration (a change in psychology and behavior) comes from interactions with other cultural groups. It reflects the conflict of different cultures at an individual level. During the process of urban integration, the psychological integration of migrant workers is an essential and ultimate stage in migrant workers becoming urban citizens [25].

A change in migrant workers’ perceptions of happiness is a direct reflection of whether they have a stable life. Happiness, first explored from the philosophical perspective, is expressed in Latin as “beatitudo”, meaning blessing. At that time, the sense of happiness was focused more on the description of this psychological state and its causes. Easterlin initially introduced the concept of happiness into economic research by creating the cross-study field called “Happiness Economics” [26], which has two streams of research. The first stream focuses on the relationship between subjective perception and utility level, the happiness measurement, and the related concepts and theories of happiness [27]. The second stream examines the influencing factors of subjective happiness, including social system conditions, economic factors, population, and other social factors. The measurement of happiness and the interpretation of results are major parts of Happiness Economics research.

**Figure 1:**
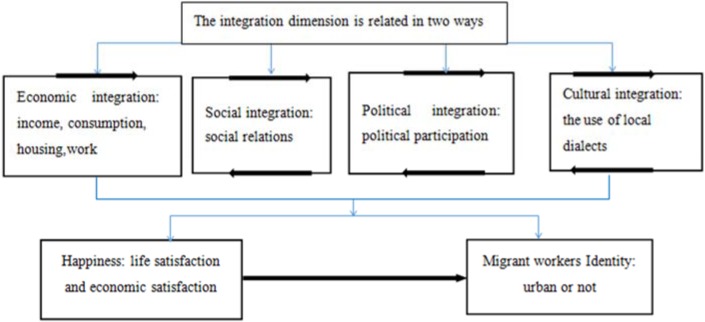
A theoretical model of urban integration and happiness of migrant workers.

Figure 1 is a theoretical model showing migrant workers’ urban integration and happiness. Though there are many studies on Happiness Economics, their main focus is explaining the source and determinants of residents’ happiness. Little research is dedicated to the effect of happiness on the psychological perception and behaviors of the residents. Research on urban integration of migrant workers is concentrated on the aspects of economic and social integration. This paper, which provides a new perspective in its examination of the impact of happiness on psychological integration during the process of migrant workers’ urban integration, will enrich the field of Happiness Economics because the sample it uses is from China, a country with a large number of migrant workers.

## 3. Hypothesis Development

Urban integration is often associated with lower integration pressure, stronger subjective happiness, more active pro-social and economic behaviors [28,29], and better school adaptations [30]. When migrant workers arrive in a new city, they suffer from a variety of psychological disorders that will seriously affect their psychological integration, such as the dilemma between employment back in their hometown versus settlement in the city, pessimism due to the struggles in the urban underclass, and psychological deprivation resulting from institutionalized discrimination and a lack of a sense of belonging. Psychological factors have an important impact on the subjective happiness of the migrating population. The existing literature, however, pays attention to only those factors that determine the happiness of residents, while ignoring the influence of happiness on personal behaviors and psychological perception.

Happiness is closely related to emotions. In general, happier people tend to have more positive emotions (e.g., joy, satisfaction, pleasure, self-esteem, and self-confidence) and fewer negative emotions (e.g., sadness, depression, and calmness) [31,32]. Social psychology research shows that emotions have both positive and negative significant effects on the perception, judgment, and behavior of individuals. Positive emotions can promote the perception and judgment of individuals in a more active manner, thus broadening the field of perception. Positive emotions also help individuals to choose more comprehensive, flexible, and creative thinking methods so as to find “pleasure” more easily [33]. Negative emotions result in deviations in the perception and judgment of individuals [34], therefore limiting the field of perception. Moreover, this impact can be long-lasting [33]. Happiness is relatively stable and free from great fluctuations caused by short-term emotional changes [35]. Research also finds that happiness can generate differences in psychological perception and judgment between individuals [36]. Specifically, happier people are more optimistic about events that have occurred [37]. They evaluate people around them as being more friendly [38] and feel less discrimination from local residents [39,40]. In addition, happier people are more concerned about their local society, and they have more affection for and a stronger willingness to integrate into their local society; as a result, happier people can be more easily accepted by locals [41].

Happiness can also lead to differences in individuals’ behaviors. Positive emotions make people succeed more easily. Negative emotions, on the one hand, have negative effects on individuals; on the other hand, they make individuals generate the urge to work hard to adjust the action strategy quickly [35]. Research has shown that people with a higher level of happiness are friendlier to those around them. Further, they take the initiative to help others or become generous economically [39]. De Neve and Oswald [42] find that happiness can improve both personal creativity and labor productivity, while Gielen and Ours [43] find that happiness can also significantly improve job searching efforts. Happiness may also increase the social capital of individuals, such as having more trust in others and more willingness to participate in elections, as well as more frequent participation in social gatherings, voluntary activities, and cultural and religious activities. Based on these studies, we develop our first hypothesis.

**Hypothesis** **1** **(H1).** Happiness has a positive impact on the urban psychological integration of migrant workers.

Bradburn [44] proposes a model to evaluate happiness, based on emotional orientation. He believes that happiness includes both positive and negative emotions, which are mutually independent. Happiness is the balance between positive and negative emotions. Diener [45] further puts forward the concept of subjective happiness, which has three main components: personal cognition (such as life satisfaction or marital satisfaction), pleasant emotions (such as pleasure), and unpleasant emotions (such as depression) that people experience. Of these, as a cognitive factor, life satisfaction is a factor independent from emotions and a key indicator for measuring subjective happiness. Life satisfaction can be subdivided into overall life satisfaction (e.g., satisfaction with the current, past, and future life) and satisfaction in special life areas (e.g., work, family, health status, economic status, ego, and group belongingness).

Life satisfaction is individuals’ comprehensive cognitive judgment on their own lives, which is an important part of happiness [46]. Migrant workers’ satisfaction with urban life is intimately bound up with their political participation, their community attachment, and their identification. The more migrant workers are satisfied with urban life, the more they are satisfied with family ties, social interaction, and working conditions in the city. Life satisfaction is another factor that affects the integration of migrant workers into urban life [47]. In the context of urbanization and industrialization, the new generation of migrant workers generally maintains a positive attitude towards the present life situation and is more willing to integrate into urban life [48]. In essence, migrant workers’ integration into urban living is a continuous improvement process of their life satisfaction in the city. Whether migrant workers can take on the identification of “city dwellers” largely depends on their recognition of their own life satisfaction. Hence, we develop our next hypothesis.

**Hypothesis** **1a** **(H1a).** Life satisfaction has a positive impact on the urban psychological integration of migrant workers.

Happy people tend to participate more frequently in social activities and gatherings. The economic satisfaction of migrant workers is closely related to their working and living conditions, their community attachment, and their urban identification. The 2015 Nationwide Migrant Worker Monitoring Survey Report (National Bureau of Statistics of China) shows that the per capita monthly incomes of migrant workers (3359 Yuan) and of local peasant workers (2781 Yuan) were both far below the average monthly income of urban residents. Migrant workers’ full recognition of socioeconomic satisfaction, as an essential social psychological mechanism, is considered to be a significant indicator of their urban integration. Economic satisfaction is the recognition of, satisfaction with and attachment to the city, based on life and labor employment, income and the material standard of living, the potential development opportunities and the available reserve of social resources, as well as other objective material conditions. Individuals may be affected by the relative economic difficulties subjectively perceived [49]. An increase in the economic status of individuals might lower the native residents’ exclusion of immigrants [50]. Thus, we develop the following hypothesis.

**Hypothesis** **1b** **(H1b).** Economic satisfaction has a positive impact on the urban psychological integration of migrant workers.

Most empirical studies use self-assessments to measure happiness. To more comprehensively reflect the real-life status of migrant workers, it is necessary to adopt the measurement indices of social integration with their multiple dimensions of economy, society, politics, culture, and identity at the same time [51]. The urban integration of migrant workers is their process of adaptation to urban life and the continuous improvement process of their life satisfaction through their interactions with local residents. Although there is a certain order among the various integration dimensions, interdependence and reciprocal causation are more important [52]. Cultural integration includes adapting to the modern industrial culture and the regional culture flowing into the city [53]; it mainly involves the transition of migrants in language, daily habits, norms, and other aspects, which is reflected in their clothing, emotional expressions, values, and behaviorial habits. It is a key entry point that they are first confronted with in urban integration [54]. For economic integration, which provides a basis for their survival and their foothold in the city, the measurement indices usually include occupation, income, and possession of real estate property. Social integration emphasizes the integration in social capital and social interaction [25]. Achieving economic integration is the most critical and important step in the process of urban integration for migrants [55]. Compared with the previous aspects, psychological integration is the highest level of integration. The success of urban integration can be considered as being built on a high level of psychological identification of immigrant areas [56]. Therefore, theoretically, the realization of integration from cultural and socioeconomic perspectives will help achieve their integration at the psychological level [6,10,21]. In short, the economic, social, political, and cultural integration of migrant workers may have an impact on their psychological integration.

The exclusiveness of urban culture and the increase in living costs have become two main obstacles to this integration. When individuals make judgments on happiness, they will inevitably combine the imprint of their culture. The process of urban integration of migrant workers is also the continuous construction and improvement of their happiness in the city. Economic pursuit serves as an important driving force and goal for migrant workers to work far away from their native homes. The more satisfaction the migrant workers have with their incomes, the higher their happiness will be [57]. Signing labor contracts and participating in social insurances are conducive to elevating the urban life happiness of migrant workers [58]. When migrant workers have access to relatively stable occupations and income, their professional appeals will no longer be confined to low-level economic gains and losses. Instead, they are gradually moving to advanced level of demands, such as better working environments, more rights protection, and psychological authorization [59,60,61,62,63,64,65,66]. These factors, therefore, will directly affect the happiness of migrant workers. The social networks and social capital, as well as other informal systems, are the main ways for migrant workers to integrate into urban society. Their integration process involves complex social factors such as social services, social interactions, and broad social rights. Social engagement and community activities have narrowed the social distance [67,68,69,70,71,72]. More migrant workers turn to communities closer to where urban residents live so as to improve their perception and satisfaction with the urban life [47]. Accordingly, Hypothesis 2 is assumed.

**Hypothesis** **2** **(H2).** Economic, social, political, and cultural integration affects the urban psychological integration of migrant workers through happiness.

## 4. Data Source and Research Design

### 4.1. Data Source

To estimate the influence of happiness on the psychological integration of migrant workers, we employ the 2014 data of China Labor-Force Dynamics Survey (CLDS) provided by the Center for Social Survey at Sun Yat-sen University in China. CLDS carried out the first formal investigation in 2012, with visits to 303 villages, 10,612 families, and 16,253 individuals in China. Their 2014 follow-up survey and supplementary investigation, based on the samples of 2012, added 101 communities, randomly selected from the sampling box produced by the map address method, making it a survey of 404 communities in 34 provinces in China, covering 15,000 family samples and 23,595 individual samples. The main objects surveyed by CLDS are labor force aged from 15 to 64, as well as workers aged 65 or more, and a representative sample covers a wide range of aspects including work, health, psychology, and other aspects. Our sampling method uses multi-stage and multi-level probability sampling in proportion to the size of the labor force. We take migrant workers as our research object. Our sample ends with a total of 257 communities and 1625 individuals from 29 provinces in China.

### 4.2. Model Specification

This study focuses on the examined influences of happiness of migrant workers on their psychological integration. We start with a simple regression based on the equation below:

(1) $$PSI_{i}=\beta_{0}+\beta_{1}X_{i}+\beta_{2}EI_{i}+\beta_{3}SI_{i}+\beta_{4}PI_{i}+\beta_{5}CI_{i}+\beta_{6}Control_{i}+\varepsilon_{i}$$

Which the dependent variable PSI denotes psychological integration, and the independent variable X represents subjective happiness, life satisfaction, or economic satisfaction. EI represents the economic integration of migrant workers, SI is the social integration, PI is the political integration, and CI is the cultural integration. In addition, we add a series of control variables, Control, which may influence the psychological integration of migrant workers. ε is a random disturbance term (see Table 1 for details).

In order to verify the robustness of the happiness regression results, we use the stepwise regression method first, followed by Equation (2), and then the variables of economic, social, political, and cultural integration are gradually added according to Equation (1).

(2) $$PSI_{i}=\beta_{0}+\beta_{1}X_{i}+\beta_{6}Control_{i}+\varepsilon_{i}$$

The dependent variable of psychosocial integration in Equation (1) may have an effect on happiness. There may also be some unobservable factors, such as unobserved personal available resources, which can affect both psychological integration and happiness. Particularly in cross-sectional data, these unobservable factors may cause a misunderstanding that happiness is indeed highly associated with psychological integration. These questions would lead to the endogeneity issue in empirical analysis. An effective way to overcome the endogeneity issue is to find a valid instrumental variable (IV) for endogenous variables. Liu [60] recommended the construction of an instrumental variable as follows: select the mean value of the happiness of others (we construct the instrumental variables of life satisfaction and economic satisfaction by using the equation consistent with happiness) in the community, excluding the migrant workers, as the instrumental variable for happiness. The instrumental variable is thus specified as follows:

(3) $$Happiness\_iv_{i}=\underset{j\neq i}{\sum }Happiness_{i}/\left(n-1\right)$$

We also examine the impact of other integration dimensions on psychological integration through the happiness, life satisfaction, and economic satisfaction of the migrant workers. The model is specified as follows:

(4) $$X_{i}=\beta_{0}+\beta_{1}EI_{i}+\beta_{2}SI_{i}+\beta_{3}PI_{i}+\beta_{4}CI_{i}+\beta_{5}Control_{i}+\varepsilon_{i}$$

In Equation (4), the explained variable X represents subjective happiness, life satisfaction, and economic satisfaction. The definitions of EI, SI, PI, CI, and Control are the same as above. In this paper, Stata (StataCorp LLC, College Station, TX, USA) is used for the regression analysis, and AMOS (Advanced Mechanical and Optical Systems, Liege, Belgium) is used for the structural equation estimation of the model.

## 5. Definition for and Summary Statistics of Each Variable

Table 1 provides the detailed definition and summary statistics of each variable.

**Dependent Variables.** Psychological integration includes two dimensions, settlement willingness, and trust level. (1) *settlement willingness*: Proportions of migrant workers who tend to stay in the employment place, who do not tend to stay in the employment place, and who are uncertain about their decision are 39.2%, 40.31%, and 20.49%, respectively; (2) *trust level*: 67.2% of migrant workers do not trust or are not sure whether they trust other residents in the same community.

**Independent Variables.** Happiness is the key variable measured in our study. We conduct a systematic measurement of happiness from three main aspects: happiness, life satisfaction, and economic satisfaction. As psychological integration is affected by other factors, we also introduce variables such as economic, social, political, and cultural integration.

**(1) Sense of Happiness.** The average scores of happiness, life satisfaction, and economic satisfaction of migrant workers are 3.62, 3.47, and 3, respectively, which lie between “happy” and “neither good nor bad”. Moreover, life satisfaction is significantly superior to economic satisfaction.

**(2) Economic Integration.** We measure economic integration from three perspectives: (a) *working conditions*, including incomes and working hours. In 2013, the average gross income of migrant workers is 44,522 Yuan per annum, and they worked for nearly 54 h for a recent week in 2014; (b) *social security situation*, including employee medical insurance, resident health insurance, employee endowment insurance, retirement pension, employment injury insurance, maternity insurance, and unemployment insurance. The proportions of migrant workers participating in urban employee medical insurance and urban resident medical insurance are 17.85% and 6.89%, respectively. The proportions of migrant workers participating in urban employee endowment insurance and urban resident endowment insurance are 12.43% and 3.08%, respectively. The proportion of migrant workers paying the housing provident fund is 9.97%. The proportions of employment injury insurance, maternity insurance, and unemployment insurance are 17.72%, 9.48%, and 12.98%, respectively. The percentages above indicate that migrant workers often do not obtain sufficient social security; (c) *human capital*, measured by indicators of vocational training. 12.12% of migrant workers participate in vocational training, indicating that migrant workers have few opportunities to achieve professional training, resulting in the slow accumulation of human capital.

**(3) Social Integration.** We measure social integration from two perspectives: (a) *Social networks*, including two dimensions: the number of local friends and the support from local friends. Migrant workers, on average, have 9 friends in the local area. Within these local friends, the number of friends to whom migrant workers can talk about their inner feelings, discuss issues, and obtain financial aid (5000 Yuan as the standard) is reduced to 3; (b) *community interactions*, including two dimensions: community familiarity and community mutual assistance. The average value of migrant workers’ community familiarity is 2.99, and the average value of community mutual assistance is 2.67, which are between “general” and “not very good”.

**(4) Political Integration**. We measure political integration from two aspects: (a) *community votes*, The proportion of migrant workers who ever participated in community elections is 11.57%, indicating that the right to vote and stand for election of migrant workers has basically been a mere formality, and migrant workers cannot effectively participate in public policy making in the immigrant area; (b) *social organizations*, the proportion of migrant workers who participate in social organizations is 36.37%, indicating that migrant workers cannot effectively maintain their own interests through social organizations.

**(5) Cultural Integration**. Cultural integration is measured from two perspectives: (a) *modernity*, including three dimensions: the use of mobile phones, online banking, and the support of online ticketing. The proportions of migrant workers are 79.02%, 42.65%, and 40.92%, respectively; (b) *language integration*, which is measured by the use of dialects. Among them, migrant workers who cannot speak dialects account for 18.22%; those who can only speak a little bit account for 9.97%, those who master partial dialects account for 6.52%, those who master the majority of dialects account for 16.74%, and those who fully master the dialects account for 48.55%.

In order to control the influence of individual characteristics on the psychological integration of migrant workers, we use eight control variables in the model: age, age squared, gender, party members, education level, working type, religion, and marital status. The average age of migrant workers is 37, among which male migrant workers account for 44.12%, and migrant workers with no more than a high school education account for 89.29%. Employees, employers, self-employed people, and farmers account for 52.86%, 2.46%, 15.02%, and 3.94%, respectively. Migrant workers with acknowledging a religion account for 9.72%. The proportions of migrant workers who are unmarried, newly married, remarried, divorced, widowed, or cohabiting are 16.18%, 78.15%, 2.15%, 1.35%, 0.68%, and 1.48%, respectively.

## 6. Empirical Results

### 6.1. Main Regression Results

After the indexes included in each dimension are determined, we follow Nie and Feng [73,74] to use exploratory factor analysis and the weighted average method to reduce and integrate various factors of the integration. We extract common factors from the indexes of economic integration, social integration, and cultural integration by using principal component analysis; we also conduct orthogonal rotation on the factor loading by using the variance maximization method. We find that there are two common factors in economic integration and one common factor in social integration and cultural integration. The values of the Kaiser-Meyer-Olkin statistic are 0.817, 0.733, and 0.641, respectively. The *P*-value of Bartlett’s test of sphericity is 0.000. The score of the total factor of economic integration, social integration, and cultural integration can be obtained by adding the values achieved by multiplying the factor score values extracted by the variance contribution rates. The political integration and psychological integration have gone through dimension-reduced processing by using the weighted average method.

Before conducting the two-stage least squares estimation, it is necessary to check the endogeneity of the independent variables. In addition, the underidentification test and the weak instrumental variable test need to be conducted for the instrumental variables. The results are shown as follows: (1) The Durbin-Hausman test results show that happiness, life satisfaction, and economic satisfaction are endogenous variables; (2) The underidentification test rejects the original hypothesis that the instrumental variables are endogenous; (3) The weak instrumental variables test does not reject the original hypothesis that there is a strong correlation between the instrumental variables and endogenous explanatory variables. Therefore, we use a two-stage least squares estimation to estimate the model. We calculate the variance inflation factor between various independent variables. The results show that the values of other variables are less than 10, except for ages and age squared, indicating that there is no multi-collinearity issue.

Table 2 shows the regression results. From this table, the results of Model (1) are clear that when individual characteristics are controlled, the coefficient of happiness is significantly positive at the 1% level, indicating that an increase of happiness of migrant workers will improve the level of their psychological integration. This supports our H1. The results of Model (2) show that the coefficient of life satisfaction is significantly positive at the 1% level, indicating that the increase of life satisfaction of migrant workers will improve the level of their psychological integration. It thus lends support to our H1a. The results of Model (3) show that the coefficient of economic satisfaction is significantly positive at the 1% level, indicating that an increase in the economic satisfaction of migrant workers will markedly improve the level of their psychological integration. It is therefore consistent with H1b. Comparison of the results for Models (2) and (3) shows that the coefficient of life satisfaction, 1.017, is considerably greater than the coefficient of economic satisfaction, 0.88. Obviously, at this stage, the life satisfaction of migrant workers plays a more active role during their psychological integration.

The results for Models (4–6) show that, after other dimensions of the integration are added, the significance and size of the coefficients of happiness, life satisfaction, and economic satisfaction remain similar. The coefficients of social integration and political integration are significantly positive at the 1% level. The improvement of these two integrations will significantly enhance the level of psychological integration of migrant workers, which is a direct effect. However, the coefficients of economic integration and cultural integration are not significant. For the control variables, those of working type, education level, and marital status have significant impacts on the level of psychological integration of migrant workers. Specifically, migrant workers with an education level of junior colleges or above have a higher level of psychological integration than that of migrant workers without an education; the employed migrant workers have a lower level of psychological integration than that of non-workers, the migrant farmers have a higher level of psychological integration than that of non-workers; and the remarried migrant workers have a higher level of psychological integration than that of unmarried migrant workers.

### 6.2. Initial Experience of Effects of Happiness on the Psychological Integration Mechanism

The CLDS survey data is used to further examine and identify the impact of happiness on the psychological integration of migrant workers. In accordance with the integration order, urban integration can be considered as economic integration, behavior adaptation, cultural acceptance, and identity recognition. We identify the order of migrant workers’ urban integration as economic integration, social integration, political integration, cultural integration, and psychological integration. Our empirical results find that social integration and political integration directly affect happiness. Therefore, we will empirically examine and identify the channels through which these may affect psychological integration.

#### 6.2.1. Channels of Happiness

Migrant workers leave their hometowns and work hard in the city to improve their quality of life and pursue a happier life. The high social integration indicates that migrant populations have a deep understanding of the city’s customs and habits, social affections, and surroundings. They often participate in various activities carried out by communities and by locals to seek resources and cultural identity. These resocialization processes can help migrant workers to quickly integrate into urban life, so as to achieve their upward social mobility, and thus help improve their happiness. The results for Model (1) in Table 3 shows that the coefficients of economic integration, social integration, and cultural integration are markedly positive at the 1% level of significance, indicating that economic integration, social integration, and cultural integration affect the psychological integration through the happiness of migrant workers.

#### 6.2.2. Channels of Life Satisfaction

The process of urban integration of migrant workers is the continuous improvement process of their life satisfaction in the city. Their incomes, health conditions, social statuses, social welfare, and housing have a significant impact on their life satisfaction [75,76]. Social support can provide material or informational help, increase people’s sense of joy and sense of belonging, and improve their self-esteem and self-confidence, which can be regarded as a catalyst for interpersonal relationships, to stimulate individuals to seize and create opportunities actively that will improve their enthusiasm for self-development, thereby experiencing more life satisfaction. The results for Model (2) in Table 3 show that the coefficients of economic integration, social integration, political integration, and cultural integration are positive at the 10%, 5%, or 1% level of significance, respectively. These results indicate that economic, social, political, and cultural integration affects psychological integration through the life satisfaction of the migrant workers.

#### 6.2.3. Channels of Economic Satisfaction

Higher incomes normally bring more material enjoyments, higher rights, and status, along with higher self-confidence. Clark and Oswald [75] argue that losses due to unemployment are not only reflected in incomes but also have a psychological influence, including frustration, depression, and loss of reputation. Migrant workers who establish a more harmonious interpersonal relationship by obtaining higher salaries obtain opportunities for promotion and complete human capital accumulation required by future development in their work. Through these processes, they also improve their job and economic satisfaction. The results for Model (3) in Table 3 demonstrate that the coefficients of social integration, political integration, and cultural integration are positively significant at the 1% level. This result indicates that these three factors affect psychological integration through the economic satisfaction of migrant workers.

## 7. Robustness Check

### 7.1. Intergenerational Differences

In the process of urban integration, migrant workers are no longer a homogeneous group. The new generation of migrant workers has gradually become the main force after polarization [63]. The intergenerational differences are generated due to differences in ages and growth backgrounds, with the new generation of migrant workers keeping a more positive attitude towards urban integration. They have higher pursuits of modern lifestyles and a greater willingness for citizenization. Taking into account the influence of this intergenerational difference, we further investigate the impact of happiness of migrant workers on their psychological integration by groups. The results in Table 4 show that the happiness of the new and old generations of migrant workers has significantly positive impact on their psychological integration. However, the influence of happiness of the new generation of migrant workers on their psychological integration is greater than that of the old generation of migrant workers. Life satisfaction and economic satisfaction of the old generation influence psychological integration more greatly than those of the new generation of migrant workers influence it. The results show that the happiness effect of the new generation of migrant workers is more prominent. In addition, the old generation of migrant workers’ political and cultural integration has a more significant impact on their psychological integration than the new generation has. The new generation of migrant workers focuses more on their future development opportunities; they are also more motivated. As a result, their happiness has a greater influence on their psychological integration than that of the old generation of migrant workers.

### 7.2. Self-Selection Problem

There is a problem of self-selection among migrant workers. Hence, we use the factor propensity score matching to solve this problem. The difference between the average happiness and the individual happiness of migrant workers is regarded as the treatment item. The matching analysis between treatment groups with positive differences and the control groups without differences, as well as with the control groups with negative differences, are conducted. The individual characteristic variables of migrant workers and their economic integration, social integration, political integration, and cultural integration are set to be the covariate variables. We then conduct one-to-one neighbor matching, radius matching, and kernel matching. After the matching, we find that the impacts of migrant workers’ happiness, life satisfaction, and economic satisfaction on their psychological integration are positive. Results of all three matching methods support the conclusion reached in our previous analyses. (Due to the space limitation, we do not present the results of the propensity score matching.)

### 7.3. Path Verification Test—A Structural Equation Model Analysis

In order to further observe the relationship between variables, we use the structural equation model to analyze them. Table 5 shows the evaluation and fitting index results of the structural equation estimation. As shown, the Chi-squared value of the set equation is 0.374, and the *p*-value is 0.541, so we infer that our proposed model is not rejected. In addition, the model-fitting index numbers, especially the RMSEA value of the initial model (0.001), indicate that the model fits the sample data very well.

**Figure 2:**
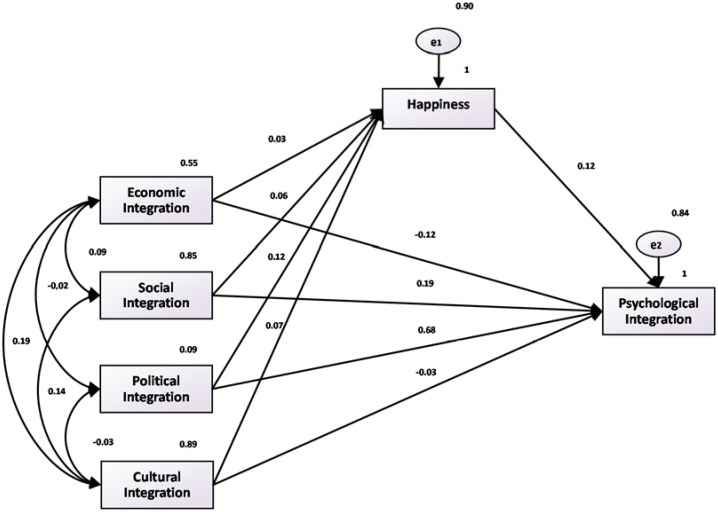
Fitting diagram of happiness and psychological integration path.

**Figure 3:**
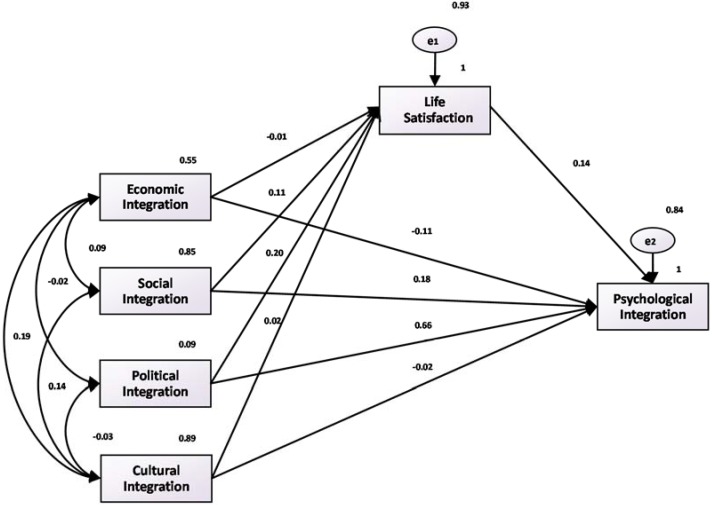
Fitting diagram of life satisfaction and psychological integration path.

**Figure 4:**
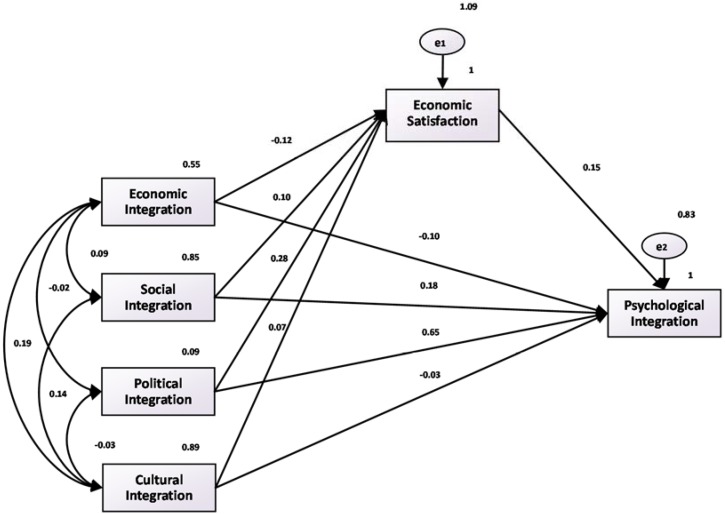
Fitting diagram of economic satisfaction and psychological fusion path.

In Figure 2, Figure 3 and Figure 4, we depict the fitting diagrams of the paths of happiness, life satisfaction, and economic satisfaction. The figures show that the coefficients of happiness, life satisfaction, economic satisfaction, and psychological fusion, which are 0.12, 0.14, and 0.16, respectively, are significant at the 1% level. These results are consistent with our earlier regression results. At the same time, this indicates that economic, social, political, and cultural integration mainly influences the integration of the mind through the direct effect. However, when economic, social, political, and cultural integration levels rise, the mediating effect of happiness also exists (i.e., through the psychological integration of happiness), which is consistent with the Table 3 regression results.

## 8. Concluding Remarks

Happier people have more positive emotions, more optimistic attitudes. They produce better expectations and get better psychological integration. This paper uses the 2014 data of the China Labor-Force Dynamics Survey (CLDS) to assess the impact of happiness on the psychological integration of migrant workers. Four interesting and important conclusions were reached.

(1) By using the instrumental variable method, we show that the improvement of the happiness, life satisfaction, and economic satisfaction of migrant workers can significantly improve their psychological integration level. Life satisfaction has a greater impact than economic satisfaction.
(2) The promotion of the social integration and political integration of migrant workers can significantly improve the level of their psychological integration. The working type, education level, and marital status of control variables have noticeable influences on the level of their psychological integration.
(3) Economic, social, and cultural integration influences psychological integration by affecting the happiness of migrant workers. Economic, social, political, and cultural integration influences psychological integration by affecting the life satisfaction of migrant workers. Social, political and cultural integration influences their psychological integration by affecting their economic satisfaction.
(4) The happiness of the younger generation of migrant workers has a greater impact on psychological integration than that of the older generation of migrant workers.

Preferential policies should thus be provided to improve the happiness of migrant workers. To improve the happiness level of migrant workers, firstly, the government should eliminate discrimination and focus on improving the institutional environment, as well as the social environment of migrant workers’ urban life, and guarantee the equal rights of citizens and their residential treatments. In more detail, it is essential for the government and local enterprises to eliminate discrimination against migrant workers in public opinions and publicity; to reduce unfair treatment and restrictions on migrant workers in the labor market; and to take appropriate measures to reduce household registration discrimination. Secondly, the government should factor the construction of a happy city and the improvement of the happiness level of residents into the evaluation of local government performance. The government needs to reduce the factors that have a negative impact on the happiness of residents, and it needs to maintain policy coherence so that the public feels confident about policies and the future. Finally, the government should enhance the education of Residents, in terms of their physical and mental health, and should popularize their mental health consulting services to improve the life satisfaction of migrant workers, thereby guiding migrant workers to develop a healthy view of happiness. Specifically, the government should improve the psychological service function of relevant institutions and should develop professional teams to engage in psychological consulting services.

From the perspective of improving the economic, social, political, and cultural integration of migrant workers, the government should emphasize the following five aspects. Firstly, the government should improve the level of social security of migrant workers. Secondly, the government needs to promote the social interactions of migrant workers, to strengthen the concept of the urban identity of migrant workers, and to promote the interactions between local residents and migrant workers. Thirdly, channels need to be established for migrant workers to express their interests easily. Fourthly, the government should establish a variety of social organizations, as well as facilitating trade unions, neighborhood committees, and community centers, which will guarantee the rights and interests of migrant workers. Lastly, the government should focus on the psychological level of migrant workers. The government should carry out multi-level, diversified cultural and recreational activities to eliminate the language barriers of migrant workers.

Our study has some limitations. Due to the nature of the data we have used, we conduct only cross-sectional data analysis; we are unable to conduct panel data analysis or to use the natural experiment method, which might yield some other explanations. Moreover, we cannot carry out a multi-level analysis, because, with the very limited number of Chinese studies in this field, it is impossible to organize an expert group to preliminarily confirm the weight value of the various indicators. We leave these issues for future research.

## Figures and Tables

**Table 1:** Variable Definition and Descriptive Statistics.

Dimension	Variables	Variable Definition	Mean	Standard Deviation	Min	Max
Happiness	Happiness	From very unhappy to very happy, numbers 1–5 are assigned to each level, respectively	3.62	0.956	1	5
	Life satisfaction	From very unsatisfied to very satisfied, numbers 1–5 are assigned to each level, respectively	3.47	0.971	1	5
	Economic satisfaction	From very unsatisfied to very satisfied, numbers 1–5 are assigned to each level, respectively	2.962	1.054	1	5
Economic integration	Income	Logarithm of the total income in 2013	7.161	4.776	0	14.91
	Working time	Logarithm of the working times in the most recent week of 2014	2.825	1.784	0	4.942
	Medical insurance	Whether attend the Urban medical insurance? 0 for no, 1 for yes	0.235	0.424	0	1
	Endowment insurance	Whether attend the Urban endowment insurance? 0 for no, 1 for yes	0.152	0.359	0	1
	Housing fund	Whether attend the housing fund or not?	0.1	0.3	0	1
		0 for no, 1 for yes				
	Work-related injury insurance	Whether attend the work-related injury insurance or not? 0 for no, 1 for yes	0.177	0.382	0	1
	Childbirth insurance	Whether attend the childbirth insurance or not? 0 for no, 1 for yes	0.095	0.293	0	1
	Unemployment insurance	Whether attend the unemployment insurance or not? 0 for no, 1 for yes	0.13	0.336	0	1
	Vocational training	1 for participating in vocational training,	0.121	0.326	0	1
		0 for no				
Social integration	Number of friends	Local friends with whom you keep regular contacts, 1 for none; 2 for 1–5 persons; 3 for 6–10 persons; 4 for 11–15 persons; 5 for 16 persons or above	2.446	1.156	1	5
	Support 1 from friends	Friends with whom you can have a heart-to heart talk, 1 for none; 2 for 1–3 persons; 3 for 4–6 persons; 4 for 6–8 persons; 5 for 9 persons or above	2.199	1.118	1	5
	Support 2 from friends	Friends with whom you can discuss issues, 1 for none; 2 for 1–3 persons; 3 for 4–6 persons; 4 for 6–8 persons; 5 for 9 persons or above	2.143	1.058	1	5
	Support 3 from friends	Friends from whom you can borrow RMB 5000 or above, 1 for none; 2 for 1–3 persons; 3 for 4–6 persons; 4 for 6–8 persons; 5 for 9 persons or above	2.067	1.162	1	5
	Community familiarity	The level that you are familiar with the inhabitants in the same community or village: five levels, from very unfamiliar to very familiar, with numbers 1~5 assigned to each level respectively	2.99	1.067	1	5
	Community mutual assistance level	The mutual assistance levels with the inhabitants in the same community or village: five types, from very low to very high, with numbers 1~5 assigned to each type respectively	2.67	1.075	1	5
Political integration	Community poll	0 for not taking part in poll; 1 for the opposite	0.116	0.32	0	1
	Social organization	0 for not taking part in; 1 for the opposite	0.364	0.481	0	1
Cultural integration	Use of mobile phone	0 for not knowing at all; 1 for knowing a little but not well; 2 for not bad; 3 for no problem	2.279	1.103	0	3
	Use of online bank	0 for not knowing at all; 1 for knowing a little but not well; 2 for not bad; 3 for no problem	1.326	1.341	0	3
	Online ticket-purchasing	0 for not knowing at all; 1 for knowing a little but not very well; 2 for not bad; 3 for no problem	1.249	1.326	0	3
	Local language integration	1 for mastering nothing; 2 for mastering a little; 3 for mastering some; 4 for mastering local dialects basically; 5 for mastering local dialects totally	3.674	1.576	1	5
Psychological integration	Settlement willingness	From very unlikely to very unlikely, numbers 1–5 are assigned to each level, respectively	3.006	1.578	1	5
	Trust level	The trust level with the inhabitants in the same community or village, which is classified into five levels, from great distrust to great trust; numbers 1–5 are assigned to each level, respectively.	3.161	0.86	1	5
Control variables	Age	Respondent’s age in 2014	37.01	12.302	15	71
	Male	0 for female; 1 for male	0.441	0.497	0	1
	Education level	1 for no school; 2 for primary school; 3 for junior middle school; 4 for senior middle school and technical secondary school; 5 for college or above	3.116	1.016	1	5
	Working type	0 for no work; 1 for employee; 2 for employer; 3 for self-employment; 4 for farmer	2.472	1.712	1	5
	Religious or not	0 for no; 1 for yes	0.097	0.296	0	1
	Marital status	1 for unmarried; 2 for newly married; 3 for remarried; 4 for divorced or widowed; 5 for cohabiting	1.966	0.731	1	6

**Table 2:** Influence of Happiness on Psychological Integration.

Variables	Psychological Integration		Psychological Integration		Psychological Integration		Psychological Integration		Psychological Integration		Psychological Integration	
	(1) 2SLS		(2) 2SLS		(3) 2SLS		(4)2SLS		(5) 2SLS		(6) 2SLS	
Happiness	0.629 ***	(0.142)					0.594 ***	(0.143)				
Life satisfaction			1.017 ***	(0.185)					0.915 ***	(0.187)		
Economic satisfaction					0.880 ***	(0.127)					0.826 ***	(0.134)
Economic integration							−0.063	(0.047)	−0.066	(0.052)	0.0296	(0.048)
Social integration							0.141 ***	(0.029)	0.091 **	(0.037)	0.115 ***	(0.033)
Political integration							0.519 ***	(0.083)	0.414 ***	(0.100)	0.350 ***	(0.100)
Cultural integration							−0.051	(0.039)	−0.064	(0.045)	−0.102 **	(0.045)
Age	0.022	(0.018)	0.041 *	(0.022)	0.031	(0.020)	0.019	(0.018)	0.035	(0.022)	0.020	(0.020)
Age squared (×1000)	−0.154	(0.213)	−0.501 *	(0.273)	−0.384	(0.246)	−0.138	(0.216)	−0.437	(0.268)	−0.289	(0.240)
Male	−0.046	(0.056)	−0.034	(0.067)	−0.081	(0.063)	−0.069	(0.056)	−0.049	(0.066)	−0.093	(0.062)
Primary school	−0.015	(0.144)	−0.101	(0.169)	−0.196	(0.172)	−0.0163	(0.138)	−0.086	(0.157)	−0.176	(0.165)
Junior middle school	0.036	(0.144)	0.002	(0.167)	−0.009	(0.168)	0.049	(0.137)	0.034	(0.154)	0.016	(0.160)
Senior middle school or Technical secondary school	0.213	(0.153)	0.252	(0.175)	0.173	(0.177)	0.264 *	(0.148)	0.327 **	(0.165)	0.230	(0.171)
College and above	0.485 ***	(0.172)	0.276	(0.207)	0.247	(0.202)	0.544 ***	(0.166)	0.408 **	(0.191)	0.337 *	(0.197)
Employee	−0.395 ***	(0.069)	−0.380 ***	(0.085)	−0.264 ***	(0.083)	−0.320 ***	(0.082)	−0.308 ***	(0.095)	−0.280 ***	(0.091)
Employer	−0.012	(0.182)	−0.279	(0.218)	−0.067	(0.204)	−0.015	(0.183)	−0.221	(0.207)	−0.069	(0.201)
Self-employment	0.008	(0.092)	0.072	(0.112)	0.164	(0.108)	0.067	(0.092)	0.118	(0.109)	0.169	(0.106)
Farmer	0.629 ***	(0.131)	0.601 ***	(0.145)	0.602 ***	(0.140)	0.495 ***	(0.127)	0.496 ***	(0.140)	0.474 ***	(0.136)
Religious or not	0.096	(0.080)	0.054	(0.096)	0.092	(0.094)	0.036	(0.077)	0.017	(0.090)	0.057	(0.090)
Newly married	−0.073	(0.133)	0.019	(0.133)	0.185	(0.118)	−0.050	(0.132)	0.050	(0.129)	0.204 *	(0.115)
Remarried	0.523 ***	(0.203)	0.662 ***	(0.226)	0.722 ***	(0.237)	0.514 ***	(0.197)	0.669 ***	(0.215)	0.732 ***	(0.228)
Divorced	0.313	(0.237)	0.555 *	(0.310)	0.661 **	(0.326)	0.332	(0.233)	0.533 *	(0.292)	0.641 **	(0.316)
Widowed	0.295	(0.299)	0.297	(0.327)	0.476	(0.441)	0.316	(0.291)	0.348	(0.316)	0.471	(0.415)
Cohabiting	−0.335	(0.222)	−0.099	(0.286)	−0.402	(0.256)	−0.277	(0.227)	−0.055	(0.280)	−0.354	(0.265)
Constant	0.366	(0.682)	−1.126	(0.848)	−0.140	(0.578)	0.391	(0.708)	−0.821	(0.863)	0.166	(0.573)
Endogeneity test (*F*) *p*	20.820	(0.000)	55.109	(0.000)	58.316	(0.000)	18.139	(0.000)	41.095	(0.000)	46.425	(0.000)
Weak instrumental variable test (*F*)	57.540		47.913		70.785		55.305		43.365		60.222	
Insufficient identification test (chi) *p*	26.770	(0.000)	43.382	(0.000)	58.592	(0.000)	25.464	(0.000)	38.209	(0.000)	49.439	(0.000)
Observations	1625		1625		1625		1625		1625		1625	

Notes: ***, ** and * represent significance levels of 1%, 5%, and 10%, respectively. 2SLS is the abbreviation of Two-stage least squares method. Underidentification test presents the result of *F*-statistic and *p*-value of Kleibergen-Paap LM, weak instrumental variable test shows the *F*-statistics of Cragg-Donald Wald, and endogeneity test presents the *F*-statistic and *p*-value of DWH. The reference item for education level is “no school”. The reference item for vocation type is “no work”. The reference item for marital status is “no marriage”.

**Table 3:** Effect of Happiness on the Mechanism of Psychological Integration.

Variable	Happiness	Life Satisfaction	Economic Satisfaction
	(1) OLS	(2) OLS	(3) OLS
Economic integration	0.112 ***	0.075 *	−0.032
	(0.037)	(0.041)	(0.043)
Social integration	0.086 ***	0.110 ***	0.094 ***
	(0.027)	(0.027)	(0.030)
Political integration	0.051	0.147 **	0.241 ***
	(0.074)	(0.075)	(0.086)
Cultural integration	0.081 **	0.066 *	0.120 ***
	(0.035)	(0.037)	(0.039)
Control variables	YES	YES	YES
Observations	1625	1625	1625
*R*-squared	0.073	0.067	0.071

Notes: ***, **, and * represent significance levels of 1%, 5%, and 10%, respectively. OLS is the abbreviation of Ordinary Least Square .The robust standard errors are shown in parentheses. Due to space constraints, the results for the control variables are not reported.

**Table 4:** Intergenerational Differences in the Influence of Happiness on Psychological Integration.

Variable	New Generation of Migrant Workers			Old Generation of Migrant Workers		
	(1) 2SLS	(2) 2SLS	(3) 2SLS	(4) 2SLS	(5) 2SLS	(6) 2SLS
Happiness	0.660 ***			0.508 ***		
	(0.192)			(0.194)		
Life		0.832 ***			0.956 ***	
Satisfaction		(0.184)			(0.335)	
Economic			0.770 ***			0.794 ***
Satisfaction			(0.144)			(0.228)
Economic	−0.088	−0.073	−0.010	−0.029	−0.097	0.056
Integration	(0.061)	(0.063)	(0.059)	(0.078)	(0.099)	(0.083)
Social	0.163 ***	0.121 **	0.134 ***	0.137 ***	0.083	0.121 ***
Integration	(0.044)	(0.051)	(0.050)	(0.039)	(0.055)	(0.042)
Political	0.377 ***	0.320 **	0.269 *	0.634 ***	0.502 ***	0.435 ***
Integration	(0.124)	(0.138)	(0.138)	(0.108)	(0.140)	(0.140)
Cultural	−0.060	−0.067	−0.131 **	−0.048	−0.105	−0.112
Integration	(0.055)	(0.061)	(0.062)	(0.059)	(0.073)	(0.069)
Control variables	YES	YES	YES	YES	YES	YES
Constant	−1.155	−2.711 *	−2.346 **	−0.212	−1.190	0.064
	(1.255)	(1.397)	(1.191)	(1.538)	(1.890)	(1.456)
Observation	763	763	763	862	862	862

Notes: ***, ** and * represent significance levels of 1%, 5%, and 10%, respectively.

**Table 5:** Evaluation and Fitting Index Results of the Structural Equation Estimation.

Index		Criteria	Output
Absolute fit measures	$\chi^{2}$	smaller is better	0.374
	GFI	>0.9	1.000
	RMR	<0.05, smaller is better	0.001
	SRMR	<0.05, smaller is better	0.0035
	RMSEA	<0.05, smaller is better	0.001
Relative fit measures	NFI	>0.9, closer to 1 is better	0.999
	TLI	>0.9, closer to 1 is better	1.024
	CFI	>0.9, closer to 1 is better	1.000
Information index	AIC	smaller is better	40.374
	CAIC	smaller is better	168.240

Notes: This table presents only the fitting index results of the happiness structural equation only. The results for life satisfaction and economic satisfaction are consistent with the happiness one. The results are available upon request from the authors.
